# Identification of Iron Metabolism-Related Genes as Prognostic Indicators for Lower-Grade Glioma

**DOI:** 10.3389/fonc.2021.729103

**Published:** 2021-09-09

**Authors:** Shenbin Xu, Zefeng Wang, Juan Ye, Shuhao Mei, Jianmin Zhang

**Affiliations:** ^1^Department of Neurosurgery, Second Affiliated Hospital, School of Medicine, Zhejiang University, Hangzhou, China; ^2^Neuro-Oncology Branch, Center for Cancer Research, National Cancer Institute, National Institutes of Health, Bethesda, MD, United States; ^3^Department of Gastroenterology Surgery, The Second Affiliated Hospital, School of Medicine, Zhejiang University, Hangzhou, China; ^4^Brain Research Institute, Zhejiang University, Hangzhou, China; ^5^Collaborative Innovation Center for Brain Science, Zhejiang University, Hangzhou, China

**Keywords:** iron metabolism, lower-grade glioma, prognosis, tumor microenvironment, bioinformatics

## Abstract

Lower-grade glioma (LGG) is characterized by genetic and transcriptional heterogeneity, and a dismal prognosis. Iron metabolism is considered central for glioma tumorigenesis, tumor progression and tumor microenvironment, although key iron metabolism-related genes are unclear. Here we developed and validated an iron metabolism-related gene signature LGG prognosis. RNA-sequence and clinicopathological data from The Cancer Genome Atlas (TCGA) and the Chinese Glioma Genome Atlas (CGGA) were downloaded. Prognostic iron metabolism-related genes were screened and used to construct a risk-score model *via* differential gene expression analysis, univariate Cox analysis, and the Least Absolute Shrinkage and Selection Operator (LASSO)-regression algorithm. All LGG patients were stratified into high- and low-risk groups, based on the risk score. The prognostic significance of the risk-score model in the TCGA and CGGA cohorts was evaluated with Kaplan-Meier (KM) survival and receiver operating characteristic (ROC) curve analysis. Risk- score distributions in subgroups were stratified by age, gender, the World Health Organization (WHO) grade, isocitrate dehydrogenase 1 (*IDH1*) mutation status, the O^6^‐methylguanine‐DNA methyl‐transferase (*MGMT*) promoter-methylation status, and the 1p/19q co-deletion status. Furthermore, a nomogram model with a risk score was developed, and its predictive performance was validated with the TCGA and CGGA cohorts. Additionally, the gene set enrichment analysis (GSEA) identified signaling pathways and pathological processes enriched in the high-risk group. Finally, immune infiltration and immune checkpoint analysis were utilized to investigate the tumor microenvironment characteristics related to the risk score. We identified a prognostic 15-gene iron metabolism-related signature and constructed a risk-score model. High risk scores were associated with an age of > 40, wild-type *IDH1*, a WHO grade of III, an unmethylated *MGMT* promoter, and 1p/19q non-codeletion. ROC analysis indicated that the risk-score model accurately predicted 1-, 3-, and 5-year overall survival rates of LGG patients in the both TCGA and CGGA cohorts. KM analysis showed that the high-risk group had a much lower overall survival than the low-risk group (*P* < 0.0001). The nomogram model showed a strong ability to predict the overall survival of LGG patients in the TCGA and CGGA cohorts. GSEA analysis indicated that inflammatory responses, tumor-associated pathways, and pathological processes were enriched in high-risk group. Moreover, a high risk score correlated with the infiltration immune cells (dendritic cells, macrophages, CD4+ T cells, and B cells) and expression of immune checkpoint (PD1, PDL1, TIM3, and CD48). Our prognostic model was based on iron metabolism-related genes in LGG, can potentially aid in LGG prognosis, and provides potential targets against gliomas.

## Introduction

Diffuse gliomas represent the most common type of primary tumor originating in the central nervous system. Oligodendrocytomas and astrocytomas, corresponding to World Health Organization (WHO) grade II and grade III tumors, are defined as lower-grade gliomas (LGGs) (1). The median overall survival (OS) time of patients with WHO II and III gliomas is 78.1 months and 37.6 months, respectively (2). Despite advances in diagnostic and treatment methods, LGG may progress into high-grade glioma in some patients, leading to reduced therapeutic responses and a poorer disease prognosis. Therefore, exploring the underlying molecular mechanisms and prognostic indicators is still urgently required for patients with LGG.

Iron, an essential dietary element, participates in both biological and pathological processes. In contrast to normal cells, many tumor cells become dependent on iron in order to grow faster and, thus, are more susceptible to iron depletion. This phenomenon is known as iron addiction (3). Data from previous studies showed that tumor cells can increase intracellular iron levels by modulating expression of the transferrin receptor, ferroportin, and ferritin (4–8). Dysregulation of iron metabolism-related genes promotes tumor cell proliferation, invasion, and metastasis (9). Iron accumulation, as well as iron-catalytic reactive oxygen/nitrogen species and aldehydes, can cause DNA-strand breaks and tumorigenesis (9, 10). Iron also participates in several types of cell death (11), especially ferroptosis (3).

The association between high-grade glioma and iron metabolism has been reported previously. Jaksch-Bogensperger et al. showed that patients with high-grade glioma have higher serum ferritin levels (12). Glioblastoma cancer stem-like cells can absorb iron from the microenvironment more effectively, by upregulating their expression levels of ferritin and transferrin receptor 1 (8). In addition, iron accumulation promotes the proliferation of glioma cells (13). Hypoxia-induced ferritin light chain expression is also involved in the epithelial-mesenchymal transition (EMT) and chemoresistance of high-grade glioma (14). Recently, some therapeutic methods targeting cellular iron and iron-signaling pathways have been tested, including iron chelation, treatment with curcumin or artemisinin, and RNA interference (10). However, the toxicities and side effects of iron chelators limit their applications in treating gliomas (15). Therefore, there is still a need to attain a deeper understanding of iron metabolism in LGGs.

In this study, iron metabolism-related genes were investigated. We performed a comprehensive bioinformatics analyses based on gene-expression levels, DNA methylation, copy-number alteration patterns, and clinical data from The Cancer Genome Atlas (TCGA). By identifying dysregulated iron metabolism-related genes, we constructed a risk-score system of LGG and validated it in the TCGA and Chinese Glioma Genome Atlas (CGGA) datasets. In addition, function analysis and gene set enrichment analysis (GSEA) were performed between the high-risk and low-risk groups to investigate the potential pathways and mechanisms related to iron metabolism. Our results showed that a 15-gene signature could be used as an independent predictor of OS in patients with LGG.

## Materials and Methods

### Assembling a Set of Iron Metabolism-Related Genes

Iron metabolism-related genes were retrieved from gene sets downloaded from the Molecular Signatures Database (MSigDB) version 7.1 (16, 17), including the GO_IRON_ION_BINDING, GO_2_IRON_2_SULFUR_CLUSTER_BINDING, GO_4_IRON_4_SULFUR_CLUSTER_BINDING, GO_IRON_ION_IMPORT, GO_IRON_ION_TRANSPORT, GO_IRON_COORDINATION_ENTITY_TRANSPORT, GO_RESPONSE_TO_IRON_ION, MODULE_540, GO_IRON_ION_HOMEOSTASIS, GO_CELLULAR_IRON_ION_HOMEOSTASIS, GO_HEME_BIOSYNTHETIC_PROCESS, HEME_BIOSYNTHETIC_PROCESS, GO_HEME_METABOLIC_PROCESS, HEME_METABOLIC_PROCESS, HALLMARK_HEME_METABOLISM, and REACTOME_IRON_UPTAKE_AND_TRANSPORT gene sets. We also reviewed the literature and added the previously reported genes (18, 19). After removing overlapping genes, we obtained an iron metabolism-related gene set containing 527 genes.

### Datasets and Data Processing

Gene expression data for 523 LGG samples (TCGA) and 105 normal cerebral cortex samples (GTEx project) were downloaded from a combined set of TCGA, TARGET, and GTEx samples in UCSC Xena (https://tcga.xenahubs.net). Clinical information for patients with LGG was obtained from using the “TCGAbiolinks” package written for R (20–22). Gene expression data and clinicopathological information for 443 patients with LGG were retrieved from CGGA database (http://www.cgga.org.cn/) and were selected as a test set. Data from patients without prognostic information were excluded from our analysis. Ultimately, we obtained a TCGA training set containing 506 patients and a CGGA test set with 420 patients. Ethics committee approval was not required since all the data were available in open-access format.

### Differential Analysis

First, we screened out 402 duplicate iron metabolism-related genes that were identified in both TCGA and CGGA gene expression matrixes. Then, differentially expressed genes (DEGs) between the TCGA-LGG samples and normal cerebral cortex samples were analyzed using the “DESeq2”, “edgeR” and “limma” packages of R software (version 3.6.3) (23–26). The DEGs were filtered using a threshold of adjusted *P*-values of < 0.05 and an absolute log_2_-fold change >1. Venn analysis was used to select overlapping DEGs among the three algorithms mentioned above. Eighty-seven iron metabolism-related genes were chosen for downstream analyses. Additionally, functional enrichment analysis of selected DEGs was performed using Metascape (https://metascape.org/gp/index.html#/main/step1) (27).

### Constructing and Validating the Risk-Score System

Univariate Cox proportional hazards regression was performed for the genes selected for the training set using “ezcox” package (28). *P* < 0.05 was considered to reflect a statistically significant difference. To reduce the overfitting high-dimensional prognostic genes, the Least Absolute Shrinkage and Selection Operator (LASSO)-regression model was performed using the “glmnet” package (29). The expression of identified genes at protein level was studied using the Human Protein Atlas (http://proteinatlas.org). Subsequently, the identified genes were integrated into a risk signature, and a risk-score system was established according to the following formula, based on the normalized gene expression values and their coefficients. The normalized gene expression levels were calculated by TMM algorithm by “edgeR” package.

$$Risk\;score=\sum_{i=1}^{n}expr_{genei}\times coeffieicent_{genei}$$

The risk score was calculated for each patients with LGG in this study, and the distribution and receiver operating characteristic (ROC) curve were plotted using “timeROC” package (30). According to the median risk score in the training set, patients were divided into high- or low- risk groups. Patients were also divided into subgroups according to clinicopathological features, including age, gender, WHO grade, histological type, isocitrate dehydrogenase 1 (*IDH1*) mutation status, 1p19q codeletion status, and O^6^‐methylguanine‐DNA methyl‐transferase (*MGMT*) promoter methylation status. Boxplot were plotted using the “ggpubr” package to identify associations between risk scores and clinical features. In addition, the relationships between risk scores and OS rates in different groups and subgroups were evaluated by Kaplan-Meier survival analysis and log-rank testing.

### Development and Evaluation of the Nomogram

To evaluate whether the risk score system can serve as an independent predictive index, univariate and multivariate Cox regression analyses were performed with clinicopathological parameters, including the age, gender, WHO grade, *IDH1* mutation status, 1p19q codeletion status, and *MGMT* promoter methylation status. All independent prognostic parameters were used to construct a nomogram to predict the 1-, 3- and 5-year OS probabilities by the ‘rms’ package. Concordance index (C-index), calibration and ROC analyses were used to evaluate the discriminative ability of the nomogram (31).

### GSEA

DEGs between high- and low-risk groups in the training set were calculated using the R packages mentioned above. Then, GSEA (http://software.broadinstitute.org/gsea/index.jsp) was performed to identify hallmarks of the high-risk group compared with the low-risk group.

### TIMER Database Analysis

The TIMER database (http://timer.cistrome.org/) is a comprehensive web tool that provide automatic analysis and visualization of immune cell infiltration of all TCGA tumors (32, 33). The infiltration estimation results generated by the TIMER algorithm consist of 6 specific immune cell subsets, including B cells, CD4+ T cells, CD8+ T cells, macrophages, neutrophils and dendritic cells. We extracted the infiltration estimation results and assessed the different immune cell subsets between high-risk and low-risk groups (34).

### Statistical Analysis

All statistical analyses in this study were conducted using R software (version 3.6.3) and GraphPad Prism (version 8.0.2). The log-rank test was used for the Kaplan-Meier survival analysis. Hazard ratios (HRs) and 95% confidence intervals (CIs) were reported where applicable. Student’s t-test and the Kruskal–Wallis test were employed in the two-group comparisons. A two-tailed *P* value of <0.05 was considered statistically significant without specific annotation.

### Availability of Data and Materials

The data we used were retrieved from open-access databases. The majority of statistical codes are available in File S1.

## Results

### Identification of Iron Metabolism-Related Gene in Patients With LGG

**Figure 1:**
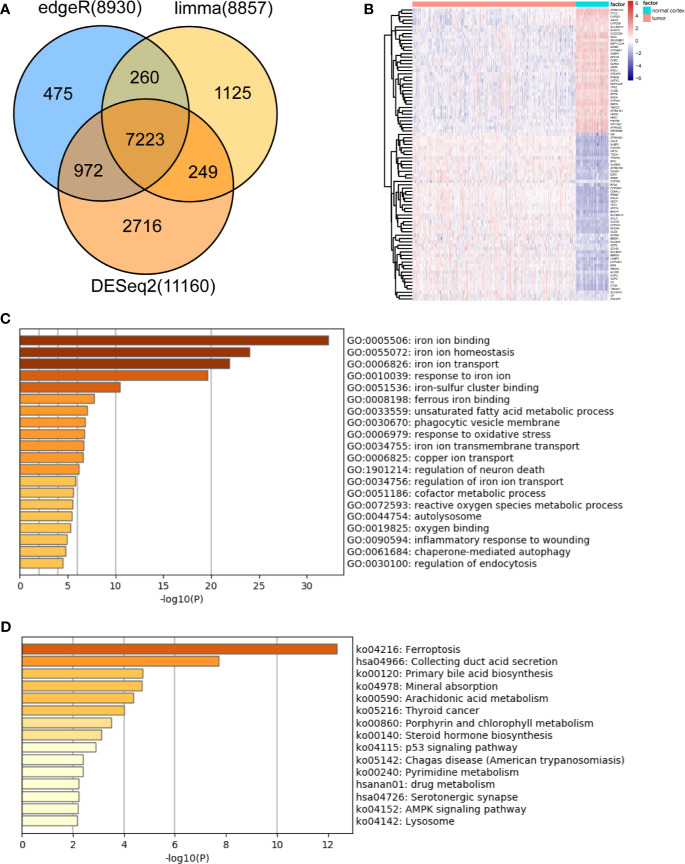
Identification and functional enrichment analysis of dysregulated iron metabolism-related genes between the TCGA-LGG cohort and normal brain cortex samples. **(A)**, Venn diagram representing intersections of DEGs identified using edgeR, limma, and DESeq2 algorithms. **(B)**, Heatmap of the expression levels of 87 DEGs related to iron metabolism. Enriched Gene Ontology terms **(C)** and KEGG pathways **(D)** associated with the 87 DEGs.

Based on the MSigDB and a literature review, we selected 527 iron metabolism-related genes for analysis. Four hundred and two genes remained after excluding genes not present in the TCGA-LGG or CGGA-LGG set. According to the criteria for DEG, we identified 7,223 DEGs between 523 TCGA-LGG samples and 105 normal brain cortex samples based on overlapping edgeR, limma, DESeq2 analysis results (**Figure 1A**). Then, a total of 87 iron metabolism-related genes (50 up-regulated and 37 down-regulated) among the DEGs were selected for further analysis (**Figure 1B**). Enrichment analyses were performed to explore the functions of the selected genes. These genes were significantly enriched in terms of iron ion binding, iron ion metastasis, and iron ion transport (**Figure 1C**). Kyoto Encyclopedia of Genes and Genomes (KEGG) enrichment analysis showed that ferroptosis, mineral absorption, the p53 signaling pathway and the AMPK signaling pathway were enriched (**Figure 1D**).

### Construction and Assessment of the Risk-Score System

**Figure 2:**
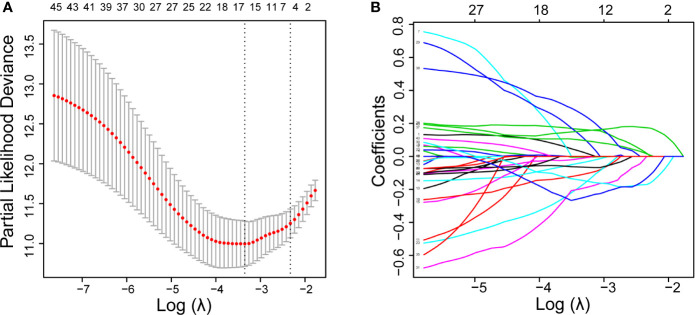
DEGs with univariate Cox regression *P*-value of < 0.05 are shown. Identification of prognostic signatures in the training set. **(A)**, Cross-validation for tuning parameter screening in the LASSO regression model. **(B)**, Coefficient profiles in the LASSO regression model.

First, univariate Cox regression was used to investigate the relationship between the expression levels of the selected genes and OS time in the training set. Using cut-off threshold of Cox *P* < 0.05, 47 genes were identified as potential risk factors related to OS (**Table S1**). Subsequently, the LASSO regression algorithm was used to refine the gene sets by calculating regression coefficients (**Figures 2A, B**). In this manner, 15 genes were identified as the most valuable predictive genes, and the risk-score system was established using the formula mentioned above (**Table 1**).

**Table 1:** Iron metabolism-related genes and their relationship with OS, and their coefficients in LASSO regression model.

Gene	Description	HR(95%CI)	*P* value	Coefficients
*ACP5*	Acid Phosphatase 5	1.19 (1.07-1.33)	0.00111	0.0287
*CH25H*	Cholesterol 25-Hydroxylase	0.893 (0.813-0.98)	0.0172	-0.039
*CYP2D6*	Cytochrome P450 Family 2 Subfamily D Member 6	0.744 (0.639-0.867)	0.000153	-0.111
*CYP2E1*	Cytochrome P450 Family 2 Subfamily E Member 1	0.685 (0.602-0.779)	9.08E-09	-0.004
*FLVCR2*	FLVCR Heme Transporter 2	0.784 (0.669-0.92)	0.00286	-0.178
*GCLC*	Glutamate-Cysteine Ligase Catalytic Subunit	0.498 (0.392-0.634)	1.46E-08	-0.012
*HBQ1*	Hemoglobin subunit theta-1	0.697 (0.605-0.804)	7.52E-07	-0.064
*KHNYN*	KH And NYN Domain Containing	2.08 (1.7-2.55)	1.76E-12	0.1640
*LAMP2*	Lysosomal Associated Membrane Protein 2	1.55 (1.14-2.11)	0.00573	0.1224
*NCOA4*	Nuclear receptor coactivator 4	0.351 (0.253-0.488)	4.69E-10	-0.194
*RRM2*	Ribonucleotide Reductase Regulatory Subunit M2	1.38 (1.25-1.52)	4.08E-10	0.099
*RTEL1*	Regulator of telomere elongation helicase 1	2.74 (1.88-3.99)	1.30E-07	0.260
*SCD5*	Stearoyl-CoA Desaturase 5	0.435 (0.349-0.544)	2.25E-13	-0.145
*STEAP3*	Six-transmembrane epithelial antigen of the prostate 3	1.67 (1.49-1.87)	1.78E-18	0.153
*UROS*	Uroporphyrinogen III Synthase	0.294 (0.213-0.405)	7.67E-14	-0.253

HR, Hazard Ratio; 95%CI, 95% confidence interval.

**Figure 3:**
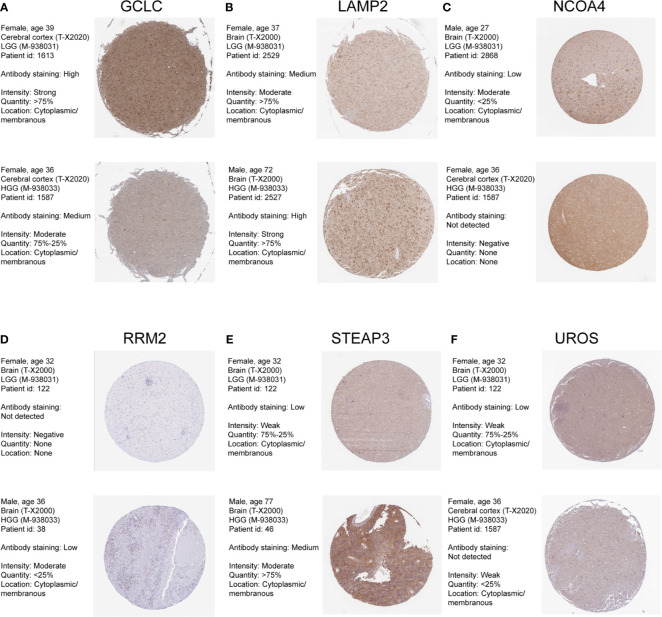
Human Protein Atlas immunohistochemical analysis of LGG and Higher-grade glioma. **(A)** GCLC; **(B)** LAMP2; **(C)** NCOA4; **(D)** RRM2; **(E)** STEAP3; **(F)** UROS.

We also confirmed the expression level of these identified genes by Immunohistochemical analysis in Human Protein Atlas (HPA). And the results were shown in **Figure 3**. 6 of these genes were dysregulated in LGG and higher-grade glioma samples. The expressions level of GCLC, NCOA4, UROS were higher in LGG samples, whereas the expression levels of LAMP2, RRM2, STEAP3 were lower in LGG than HGG samples. CH25H and RTEL1 were missing in HPA database. ACP5, CYP2D6, HBQ1, KHNYN, and SCD5 were not detected in glioma samples. However, the expression levels of CYP2E1 and FLVCR2 showed low consistency with RNA expression data.

**Figure 4:**
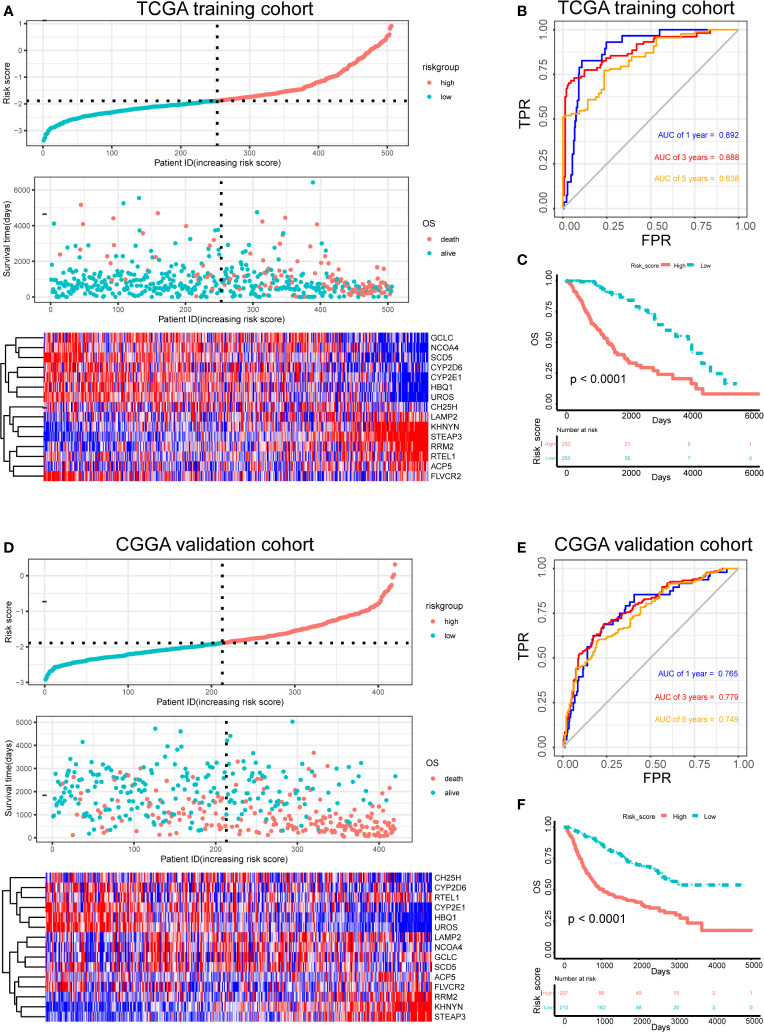
Risk score analysis, survival analysis and prognostic performance of a risk-score model based on differential expression of iron metabolism-related genes in patients with LGG. Risk score and survival time distributions, and heatmaps of gene-expression levels of the iron-metabolism signature in the TCGA **(A)** and CGGA **(D)** cohorts. ROC curves and AUC values of the risk score model for predicting the 1-, 3-, and 5-year OS times in the TCGA **(B)** and CGGA **(E)** cohorts. Kaplan–Meier survival analysis was performed to estimate the OS times between the high- and low-risk groups in the TCGA **(C)** and CGGA **(F)** cohorts.

The risk score for each patient in the training and test sets was calculated based on the expression levels of the selected genes and the regression coefficients. The distribution of risk score in training set was shown in **Figure 4A**. The median of risk score in training set was defined as threshold, which divided the patients into high-risk and low-risk groups. In addition, the distribution of survival times indicated that a higher risk score may have positively correlated with poorer outcomes (**Figure 4A**). The corresponding expression levels of the selected genes were determined (**Figure 4A**). The performance of the ROC in terms of 1-, 3-, and 5-year prognoses was analyzed (**Figure 4B**). The areas under the time‐dependent ROC curve (AUCs) were 0.892, 0.888, and 0.838, respectively, for the 1-, 3-, and 5-year OS times in the training set. Kaplan–Meier analysis and log-rank testing showed that the high-risk group had a significantly shorter OS time than the low-risk group (*P* < 0.0001; **Figure 4C**).

Furthermore, the robustness of our risk-score model was assessed with the CGGA dataset. The test set was also divided into high-risk and low-risk groups according to the threshold calculated with the training set. The distributions of risk scores, survival times, and gene-expression level are shown in **Figure 4D**. The AUCs for the 1-, 3-, and 5-year prognoses were 0.765, 0.779, and 0.749, respectively (**Figure 4E**). Significant differences between two groups were determined *via* Kaplan–Meier analysis (*P* < 0.0001), indicating that patients in the high-risk group had a worse OS (**Figure 4F**). These results showed that our risk score system for determining the prognosis of patients with LGG was robust.

### Stratified Analysis

**Figure 5:**
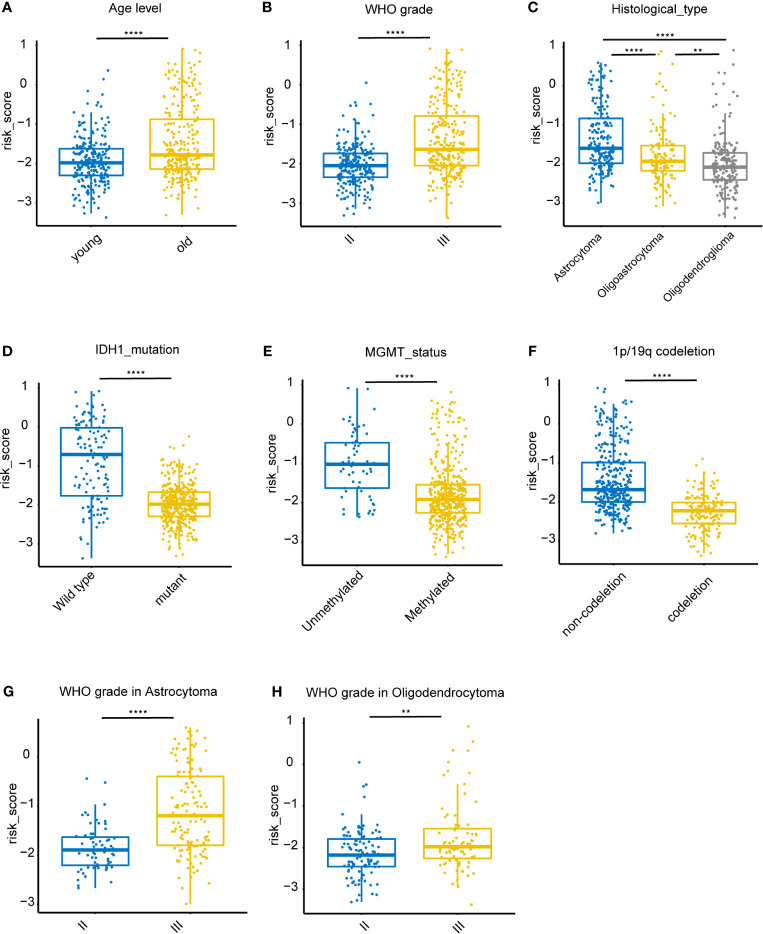
Association between clinicopathologic features and the iron metabolism based risk score in the TCGA dataset. **(A–F)**, Risk-score distributions showed statistically significant differences in LGG patients stratified by age, WHO grade, pathological types, *IDH1* mutation status, *MGMT* promoter methylation status, and 1p/19q co-deletion status. **(G)**, Distribution of risk scores between WHO II and WHO III grade in astrocytoma patients. **(H)**, Distribution of risk scores between WHO II and WHO III grade in oligodendrocytoma patients. **P < 0.005, ****P < 0.0001, ns, not significant.

Associations between risk-score and clinical features in the training set were examined. We found that the risk score was significantly lower in groups of patients with age > 40 (*P* < 0.0001), WHO II LGG (*P* < 0.0001), oligodendrocytoma (*P* < 0.0001), *IDH1* mutations (*P* < 0.0001), *MGMT* promoter hypermethylation (*P* < 0.0001), and 1p/19q co-deletion (*P* < 0.0001) (**Figures 5A–F**). However, no difference was found in the risk scores between males and females (data not shown). In both astrocytoma and oligodendrocytoma group, risk score was significantly lower in WHO II group (**Figures 5G, H**). We also validate the prediction efficiency with different subgroups. Kaplan–Meier analysis showed that high-risk patients in all subgroups had a worse OS (**Figure S1**). Besides, the risk score was significantly higher in GBM group compared with LGG group (**Figure S2**).

### Nomogram Construction and Validation

**Figure 6:**
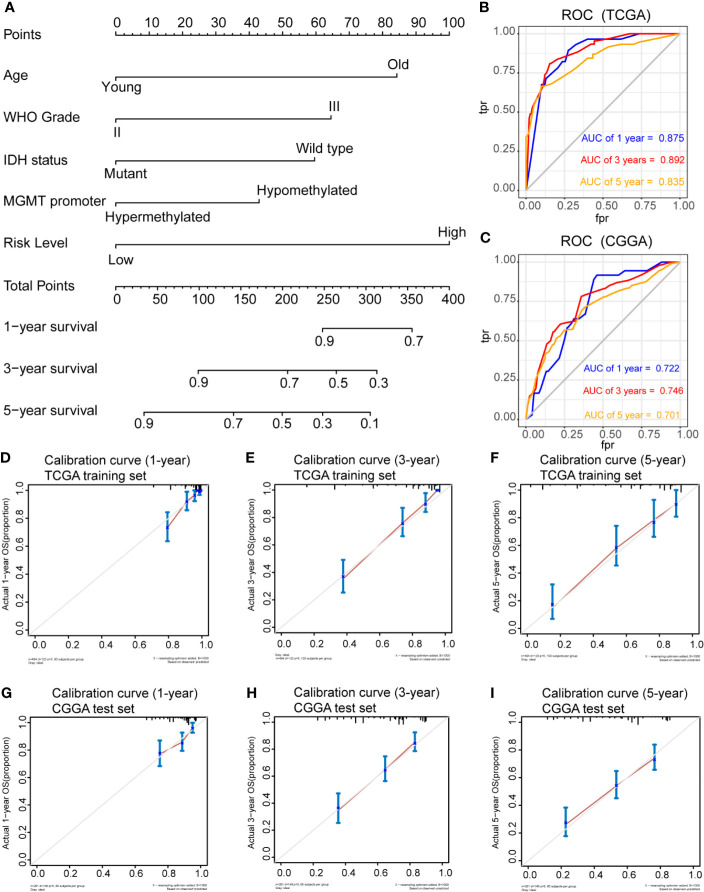
Prognostic nomogram for the 1-, 3-, and 5-year OS times of LGG patients. **(A)**, Independent risk factors screened by multivariate Cox regression in the TCGA cohort were integrated into the nomogram model. ROC curves and AUC values of the nomogram for predicting 1-, 3-, and 5-year OS in the TCGA **(B)** and CGGA **(C)** cohorts. Calibration curves of the nomogram for predicting 1-, 3-, and 5-year OS in the TCGA **(D–F)** and CGGA **(G–I)** cohorts.

To determine whether the risk score was an independent risk factor for OS in patients with LGG, the potential predictors (age group, gender, WHO grade, *IDH1* mutation status, *MGMT* promoter status, 1p/19q status and risk level) were analyzed by univariate Cox regression with the training set (**Table 2**). The individual risk factors associated with a Cox *P* value of < 0.05 were further analyzed by multivariate Cox regression (**Table 2**). The analysis indicated that the high-risk group had significantly lower OS (HR = 2.656, 95% CI = 1.51-4.491, *P* = 0.000268). The age group, WHO grade, IDH mutant status, *MGMT* promoter status and risk level were considered as independent risk factors for OS, and were integrated into the nomogram model (**Figure 6A**). The C-index of the nomogram model was 0.833 (95% CI = 0.800-0.867). Subsequently, we calculated the score of each patient according to the nomogram, and the prediction ability and agreement of the nomogram was evaluated by ROC analysis and a calibration curve. In the TCGA cohort, the AUCs of the nomograms in terms of 1-, 3-, and 5-year OS rates were 0.875, 0.892, and 0.835, respectively (**Figure 6B**). The calibration plots showed excellent agreement between the 1-, 3-, and 5-year OS rates, when comparing the nomogram model and the ideal model (**Figures 6D–F**). Moreover, we validated the efficiency of our nomogram model with the CGGA test set. The AUCs for the 1-, 3-, and 5-year OS rates with the model were 0.722, 0.746, 0.701, respectively (**Figure 6C**). The results of the calibration curves showed good agreement between the predicted OS rates and the probabilities of the 1-, 3-, and 5-year OS rates with the test set (**Figures 6G–I**).

**Table 2:** Univariate and multivariate Cox analysis of OS in TCGA-LGG dataset.

Parameters		Univariate Cox analysis		Multivariate Cox analysis	
		HR(95% CI)	*P*-value	HR(95% CI)	*P*-value
Age level	Young (≤40)	–	–	–	–
	Old (>40)	2.840 (1.940-4.150)	<0.0001	2.781 (1.837-4.210)	<0.0001
Gender	Female	–	–	–	–
	Male	1.100 (0.772-1.580)	0.589	–	–
WHO grade	II	–	–	–	–
	III	3.460 (2.330-5.140)	<0.0001	2.123 (1.394-3.232)	0.00045
*IDH1*	Wild type	–	–	–	–
	Mutant	0.287 (0.201-0.411)	<0.0001	0.525 (0.355-0.777)	0.00127
1p/19q	Non-codel	–	–	–	–
	Codel	0.378 (0.234-0.611)	<0.0001	0.666 (0.388-1.142)	0.1397
*MGMT* promoter	Unmethylated	–	–	–	–
	Methylated	0.396 (0.26-0.605)	<0.0001	0.619 (0.398-0.961)	0.033
Risk score level	Low (≤-1.8905)	–	–	–	–
	High (>-1.8905)	5.020 (3.260-7.750)	<0.0001	2.656 (1.51-4.491)	0.000268

HR, hazard ratio; 95% CI, 95% Confidence Interval.

### GSEA

**Figure 7:**
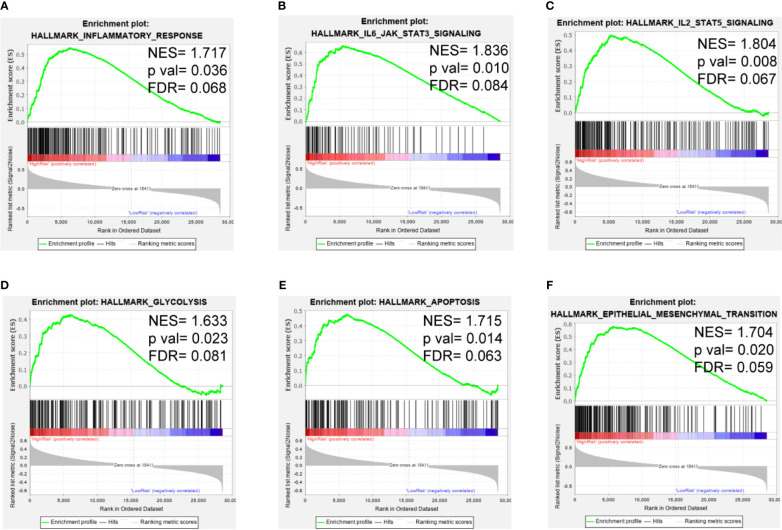
GSEA of the iron metabolism-related gene signature in the TCGA cohort. **(A–F)**, inflammatory response, IL6/JAK/STAT3 signaling pathway, IL2/STAT5 signaling pathway, glycolysis, apoptosis and the EMT were enriched in the high-risk group.

To clarify the potential impact of the expression levels of the selected genes on the LGG transcriptomic profile, GSEA analysis was performed with the high-risk and low-risk groups of the training set. GSEA revealed that several pathways, such as those related to inflammatory response, IL6/JAK/STAT3 signaling, IL2/STAT5 signaling, glycolysis, apoptosis, and the EMT, were enriched in the high-risk group (**Figures 7A–F**). These findings suggest potential roles for iron metabolism-related genes in the progression, metabolism, tumor microenvironment and immune responses of LGG.

### Immune Cell Infiltration and Immune Checkpoint Analysis

**Figure 8:**
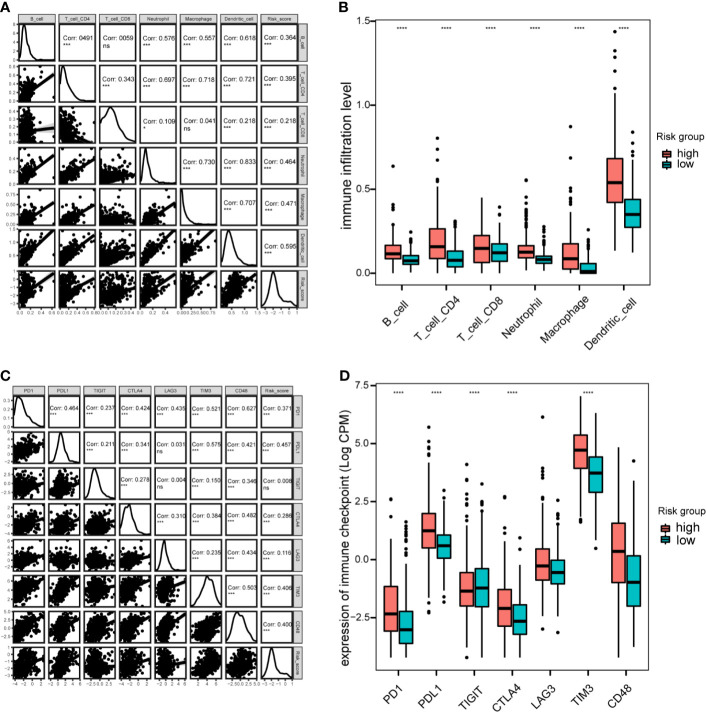
Immune cell infiltration and immune checkpoint analysis in the TCGA cohort. **(A)**, Correlation between immune cell infiltration and risk scores. **(B)**, Boxplot indicating the levels of immune cell infiltration in high-risk and low-risk LGG patients. **(C)**, Correlation matrix of seven immune checkpoint proteins and associated risk scores. **(D)**, Expression levels of immune checkpoint proteins in high-risk and low-risk LGG patients. *P < 0.05, ***P < 0.001, ****P < 0.0001, ns, not significant.

Next, the correlation between this prognostic model and the infiltration of immune cells for patients in the TCGA-LGG cohort were calculated. The proportion of different infiltrating immune cells were retrieved from the TIMER database. The results indicated that the risk score positively correlated with infiltrating immune cells, including B cells, CD4+ T cells, CD8+T cells, neutrophils, macrophages and dendritic cells (**Figure 8A**). The high-risk group showed more infiltrating immune cells, especially dendritic cells and macrophages (*P* < 0.0001; **Figure 8B**). Additionally, we assessed the relationship between risk-score model and immune checkpoint proteins (PD1, PDL1, CTLA4, LAG-3, TIM3, TIGIT and CD48). The expression levels of PD1, PDL1, CTLA4, TIM3, and CD48 positively correlated with the risk score(*P* < 0.001; **Figure 8C**). In addition, the expression levels of PD1, PDL1, and TIM3 were higher in high-risk group of TCGA-LGG cohort than in the low-risk group (*P* < 0.0001; **Figure 8D**).

## Discussion

LGG is a heterogeneous disease, especially in terms of tumorigenesis, its molecular characteristics, therapeutic responses and clinical outcomes (2, 35). Currently, recurrence or malignant progression is still inevitable, even after treatment with surgical resection, radiotherapy, chemotherapy and immunotherapy. Recently, iron metabolism was found to participate in glioma tumorigenesis, progression, and the tumor microenvironment (14, 36). GBM cancer stem-like cells uptake much more iron than non stem-like cells (37). However, the non stem-like cells have higher free iron ion level, which reduces cell viability and growth (37). Iron metabolism also recently became a therapeutic target and a potential prognostic marker of glioma (36, 38).

In this study, we used gene expression data and clinicopathological information from open-access database. Initially, we selected 87 iron metabolism-related DEGs. Among these, 15 genes were identified as potential prognostic markers by univariate Cox analysis and LASSO regression analysis, and these genes were used to construct a prognostic model. Among them, the expression levels of six genes (*RTEL1*, *KHNYN*, *STEAP3*, *LAMP2*, *RRM2*, and *ACP5*) negatively correlated with OS, whereas the expression levels of nine genes (*CYP2E1*, *GCLC*, *CH25H*, *HBQ1*, *CYP2D6*, *SCD5*, *FLVCR2*, *NCOA4*, and *UROS*) positively correlated with OS. This model was validated effective and stable with different patient cohorts, and verified as an independent predictive marker by multivariate Cox regression analysis. In addition, patients with wild type *IDH1*, *MGMT* hypomethylation, 1p/19q non-codeletion status, or a higher WHO grade had significantly higher risk scores. The higher grade gliomas contained higher proportion of stem like cells, which affected iron uptake and free iron ion level (37). Liu et al. proposed that ferritin light chain may be a upstream regulator of *MGMT* promoter methylation process (14). However, Kingsbury et al. reported that *IDH1* mutation lead to higher level of D-2-hydroxyglutarate (2HG) production, which affects the iron sensing mechanisms and promotes tumor progression (39). Variants of RTEL1 is associated with molecular subtype in IDH wild-type gliomas (32386320, 31842352). These may also result in iron metabolism dysregulation, but the underlying mechanisms still need to be further investigated.

Some data have shown that iron metabolism-related genes are involved in glioma pathological processes. *RTEL1*, an ATP-dependent DNA helicase, was reported as a risk gene for glioma (40). Some *RTEL1* variants may lead to a higher risk for glioma development (41). *STEAP3*, which encodes metalloreductase, is considered highly expressed in glioblastoma, and knocking down *STEAP3* suppresses glioma cell proliferation and metastasis (42). It was also reported that *STEAP3* drives EMT progression through STAT3/FoxM1 signaling pathway (42). *LAMP2* was found to be overexpressed in the perinecrotic areas of gliomas (43). Valdor et al. reported that *LAMP2* participated in activating chaperone-mediated autophagy in a glioma model (44). Sorafenib combined with lapatinib increased the level of LC3-GFP vesicles and reduced the level of LAMP2 (45). *RRM2* encodes the M2 subunit of ribonucleotide reductase. RRM2 was reported to promote glioma proliferation and progression through ERK1/2- and AKT- signaling pathways (46, 47). RRM2 expression induced by BRCA1, traditionally regarded as tumor suppressor, promotes tumorigenicity in GBM cells (48).

Additionally, *ACP5*, which encodes a metalloprotein enzyme, has been reported to promote tumor metastasis and recurrence in many cancers, like hepatocellular carcinoma and breast cancer (49, 50). *CYP2E1* encodes a membrane protein and is a member of the cytochrome P450 complex. *CYP2E1 Rsa*I variant has been associated with glioma (51). Bae et al. reported that inhibiting *CYP2E1* activity reduced apoptosis in glioma cells and prevented the degradation of p53 (52, 53). *CYP2D6* encodes an important member of the cytochrome P450 family. Elexpuru-Camiruaga et al. reported that the *CYP2D6* genotype correlated with the susceptibility to astrocytoma and meningioma (54). In addition, a non-functional *CYP2D6* variant was previously associated with higher recurrence rates in a breast cancer cohort (55). *GCLC* encodes catalytic subunits of glutamate-cysteine ligase, which mainly participates in glutathione synthesis and ferroptosis. Previous data showed that intratumoral thymidine from necrotic cells inhibited GCLC activity (56) and that *GCLC* expression was upregulated in *IDH1*-mutated compared to *IDH1* wild-type glioma (57). Furthermore, Yu et al. confirmed that triptolide induced GCLC degradation drove cytotoxicity due to reactive oxygen species (ROS) in *IDH1*-mutated glioma (58). The CH25H enzyme belongs to the oxidoreductase family. Previous findings showed that elevated *CH25H* expression promoted chemotactic monocyte recruitment of glioma cells (59). *NCOA4* encodes a receptor that plays important roles in ferritinophagy and iron storage. Liu et al. also identified *NCOA4* as a prognostic factor in glioma (60). COPZ1 knockdown increased the expression level of NCOA4, which elevated iron levels and reactive oxygen species, resulting ferroptosis and reduced growth of GBM cells (61). Moreover, Pinton et al. reported that *NCOA4* is overexpressed in bone marrow-derived macrophages from glioma lesions (62). *UROS*, an enzyme associated with congenital erythropoietic porphyria, participates in the heme biosynthesis pathway. Nawaz et al. demonstrated that the expression level of miR-4484, a tumor suppressor, positively correlated with *UROS* expression, which is considered the host gene of miR-4484 (63).

Some genes, like *KHNYN*, *HBQ1*, *SCD5* and *FLVCR2*, may play roles in tumorigenesis, metabolism or tumor therapy (64–68). However, the specific relationships between these genes and glioma still require further exploration.

Furthermore, we constructed a prognostic nomogram model based on iron metabolism-related genes for predicting the OS of patients with LGG. The risk score, WHO grade, and 1p/19q co-deletion status were integrated into the nomogram model. Calibration plots and ROC analysis illustrated the reliable predictive ability of the nomogram for OS with the TCGA and CGGA cohorts. This nomogram model could be used for determining patients’ prognoses and scheduling follow-up plans.

Moreover, GSEA showed that pathways associated with immune responses and tumor progression were enriched in the high-risk group. Yao et al. confirmed that activation of the IL-6/JAK/STAT3 signaling pathway led to poor outcomes in patients with glioma (69, 70). STAT5 was also found to promote glioma cell invasion (71). Both pathways are related to tumor-associated immune cells and regulate immunotherapeutic responses (72). Taga et al. reported that co-expression of genes related to the extracellular matrix, iron metabolism, and macrophages was associated with treatment outcomes in patients with glioma (36). mTOR complex 2 can control iron metabolism by regulating acetylation of iron-related genes promoter, promoting tumor cell survival (73). Previous reports showed that iron chelator therapy inhibited EMT in many cancers (74, 75). Both Dp44mT and bovine lactoferrin, as iron chelators, suppress growth, migration, and EMT process of glioma by inhibiting IL-6/STAT3 signaling pathway (38, 76). Iron complexes could suppress glioma cells proliferation associated with P53 and 4E binding protein 1 (77). Additionally, iron and copper complexes with antioxidant effects also inhibit EMT in glioma cells (78).

Immune cell infiltration analysis showed that the risk score positively correlated with the infiltration levels of immune cells, in accordance with previous data showing that higher numbers of glioblastoma-associated myeloid cells were associated with poor outcomes in GBM (79). Similarly, previous evidence suggested that M2 tumor-associated macrophages exhibited an iron-release phenotype and drove immune tolerance (9). Glioma cells could exploit monocytes as iron-string macrophages (80), and iron-related genes were overexpressed in macrophages (62). However, heme and iron can drive TAM into an proinflammatory phenotype, and iron nanoparticles are considered as promising anti-tumor agents (81). Additionally, neutrophils infiltration were induced during tumor progression(chronic ischemia, hypoxia…), resulting tumor ferroptosis and poor survival (82). Moreover, iron can modulate T cell phenotypes (83). Based on immune checkpoint analysis, our risk score also positively correlated with the expression levels of immune checkpoints proteins, like PD1, PDL1, CTLA4, and TIM3. These findings indicate that iron metabolism-related genes may predict or influence immunotherapeutic effects in patients with LGG.

## Conclusion

In conclusion, we developed and validated a risk score system based on iron metabolism-related genes from TCGA and CGGA datasets for prognosis and risk stratification. A nomogram model for 1-, 3-, and 5-year OS rate predictions was constructed and showed good predictive accuracy. The selected genes can potentially be targeted to understand the pathological mechanisms of LGG. Additionally, GSEA, tumor immune infiltration, and immune checkpoint analyses showed that iron metabolism may be involved in tumorigenesis, progression, the tumor microenvironment and immune tolerance. These results suggest promising therapeutic targets for LGG. However, large scale, prospective studies are still required to validate our model in the future.

## Data Availability Statement

Publicly available datasets were analyzed in this study. This data can be found here: https://tcga.xenahubs.net. http://www.cgga.org.cn/. Molecular Signatures Database.

## Author Contributions

XS, ZW, and JY drafted the manuscript. JZ reviewed and modified the manuscript. XS, JY, and SM revised the manuscript. All authors contributed to the article and approved the submitted version.

## Funding

This work was funded by National Natural Science Foundation of China (81701144 and 81870916).

## Conflict of Interest

The authors declare that the research was conducted in the absence of any commercial or financial relationships that could be construed as a potential conflict of interest.

## Publisher’s Note

All claims expressed in this article are solely those of the authors and do not necessarily represent those of their affiliated organizations, or those of the publisher, the editors and the reviewers. Any product that may be evaluated in this article, or claim that may be made by its manufacturer, is not guaranteed or endorsed by the publisher.

## Supplementary Material

Supplementary Figure 1 **(A–O)**, Kaplan–Meier survival analysis of the risk signature in LGG patients stratified by the age, gender, WHO grade, pathological subtypes, IDH1 mutation status, MGMT promoter methylation status, and 1p19q codeletion status.

Supplementary Figure 2 Distribution of risk scores between LGG and GBM. *****P* < 0.0001.
