# Supplementary figures and images for: Identification of Iron Metabolism-Related Genes as Prognostic Indicators for Lower-Grade Glioma
Source: Front Oncol. 2021 Sep 9;11:729103. doi: 10.3389/fonc.2021.729103 (PMC8458946; doi:10.3389/fonc.2021.729103)

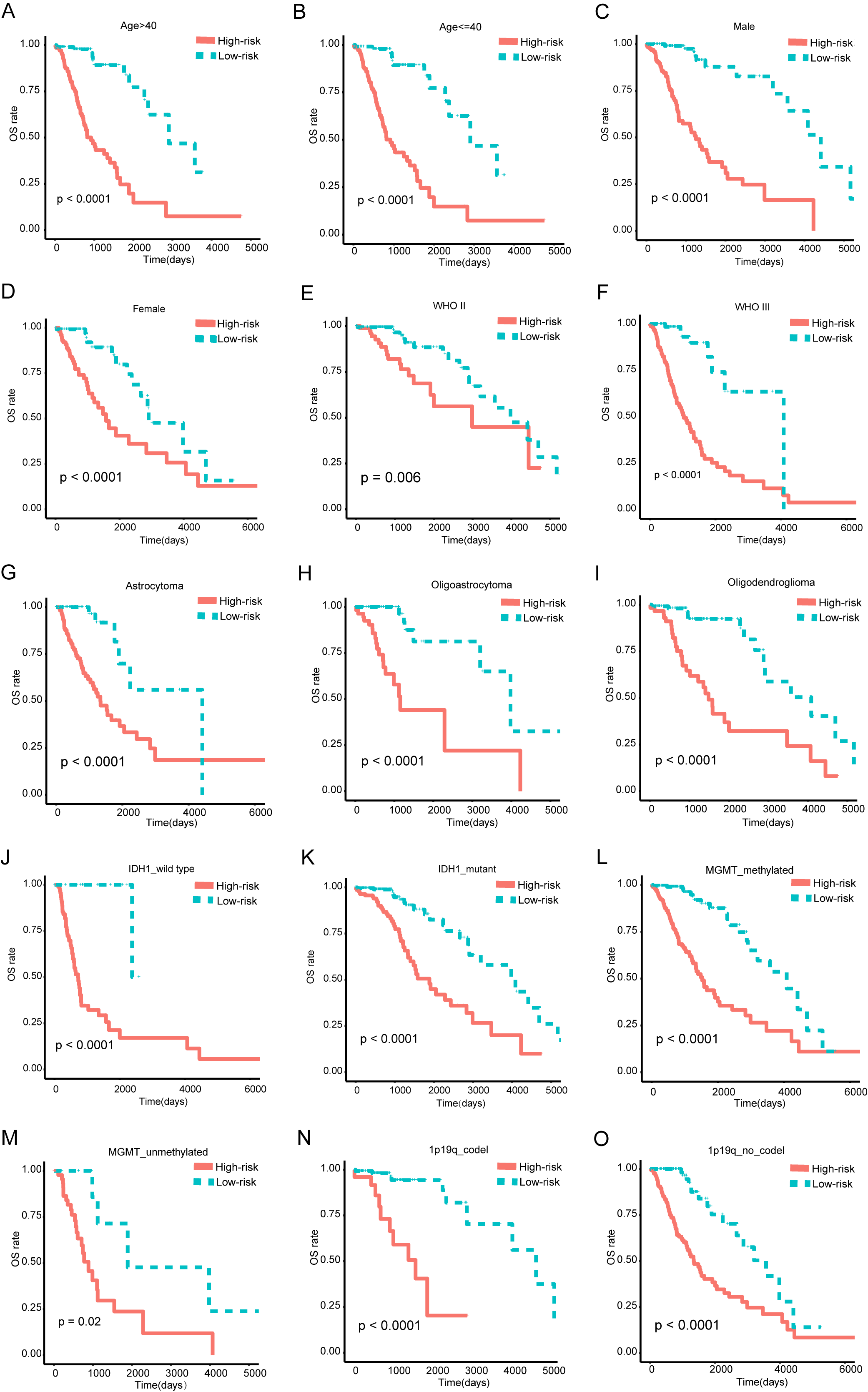

Supplement: Supplementary Figure 1 — (A–O), Kaplan–Meier survival analysis of the risk signature in LGG patients stratified by the age, gender, WHO grade, pathological subtypes, IDH1 mutation status, MGMT promoter methylation status, and 1p19q codeletion status. [file Image_1.tif]

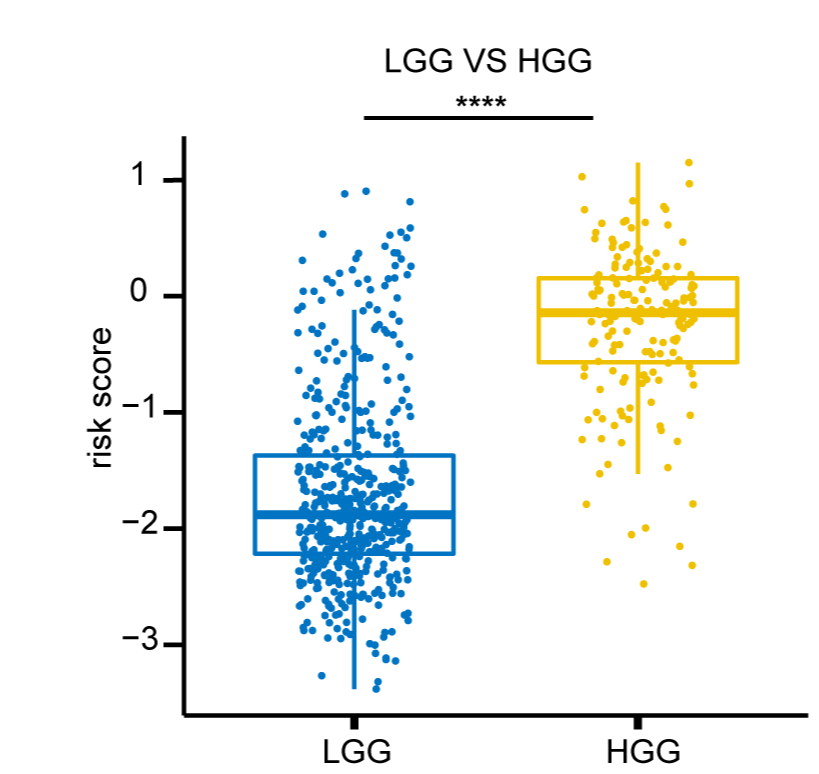

Supplement: Supplementary Figure 2 — Distribution of risk scores between LGG and GBM. ****P < 0.0001. [file Image_2.tif]
