# Supplementary material for: Identification of Iron Metabolism-Related Genes as Prognostic Indicators for Lower-Grade Glioma
Source: Front Oncol. 2021 Sep 9;11:729103. doi: 10.3389/fonc.2021.729103 (PMC8458946; doi:10.3389/fonc.2021.729103)
Supplement: Supplementary file 4 [file DataSheet_2.pdf]

# 1. Differential analysis

```
###DESeq2####
library(DESeq2)
expr = exprSet[rowMeans(exprSet)>1,]
expr<-floor(expr)
colData <- data.frame(row.names=colnames(expr),
                      group_list=group_list)
colData
dds <- DESeqDataSetFromMatrix(countData = expr,
                              colData = colData,
                              design = ~ group_list)
dds2 <- DESeq(dds)
res <- results(dds2,contrast = c("group_list","tumor","normal"))
head(res)
res <- res[order(res$padj),]
resdata <- merge(as.data.frame(res),
                 as.data.frame(counts(dds2,normalized = TRUE)),by = "row.names",sort
=FALSE)
save(resdata,file = '4 TCGA DESeq2data.Rdata')
DEG =as.data.frame(res)
DEG_DESeq2 = na.omit(DEG)

logFC_cutoff <- 1
DEG_DESeq2$change = as.factor(
  ifelse(DEG_DESeq2$padj < 0.05 & abs(DEG_DESeq2$log2FoldChange) > logFC_cutoff,
         ifelse(DEG_DESeq2$log2FoldChange > logFC_cutoff ,'UP','DOWN'),'NOT')
)
table(DEG_DESeq2$change)


#####edgeR#########
library(edgeR)
dge <- DGEList(counts=exprSet,group=group_list)
design <- model.matrix(~0+group_list)
rownames(design)<-colnames(dge)
colnames(design)<-levels(group_list)
design

keep_gene <- rowSums( cpm(dge) > 1 ) >= 2
table(keep_gene)
dge <- dge[ keep_gene, , keep.lib.sizes = FALSE ]
```

```
dge$samples$lib.size <- colSums(dge$counts)
dge <- calcNormFactors(dge)
dge <- estimateGLMCommonDisp(dge,design)
dge <- estimateGLMTrendedDisp(dge, design)
dge <- estimateGLMTagwiseDisp(dge, design)

fit <- glmFit(dge, design)
fit2 <- glmLRT(fit, contrast=c(-1,1))
DEG=topTags(fit2, n=nrow(exp))
DEG=as.data.frame(DEG)
DEG$change = as.factor(
  ifelse(DEG$FDR< 0.05 & abs(DEG$logFC) > logFC_cutoff,
         ifelse(DEG$logFC > logFC_cutoff ,'UP','DOWN'),'NOT')
)
table(DEG$change)
DEG_edgeR <- DEG


##limma ####
library( "limma" )
{
  design <- model.matrix( ~0 + factor( group_list ) )
  colnames( design ) = levels( factor( group_list ) )
  rownames( design ) = colnames( exprSet )
}
design

contrast.matrix <- makeContrasts( "tumor-normal", levels = design )

library(edgeR)
group_list = factor(group_list)
counts<-exprSet
design <- model.matrix(~0+group_list)
rownames(design) = colnames(counts)
colnames(design) <- levels(group_list)
design

dge<-DGEList(counts = counts,group=group_list)

keep_gene <- rowSums( cpm(dge) > 1 ) >= 2
table(keep_gene)
dge <- dge[ keep_gene, , keep.lib.sizes = FALSE ]
```

```
constrasts = paste(rev(levels(group_list)),collapse = "-")
cont.matrix <- makeContrasts(contrasts=constrasts,levels = design)
fit2=contrasts.fit(fit,cont.matrix)
fit2=eBayes(fit2)

DEG = topTable(fit2, coef=constrasts, n=Inf)
DEG = na.omit(DEG)
DEG$change = as.factor(
  ifelse(DEG$adj.P.Val < 0.05 & abs(DEG$logFC) > logFC_cutoff,
         ifelse(DEG$logFC > logFC_cutoff ,'UP','DOWN'),'NOT')
)
table(DEG$change)
DEG_limmavoom <- DEG

logCPM <- cpm(dge, prior.count=3, log=TRUE)
save(DEG_DESeq2,DEG_edgeR,DEG_limmavoom,group_list,file = "5 LGG_DEG.Rdata")
save(logCPM,file = “TCGA_logCPM.Rdata”)

#### DEG gene####
load('5 LGG_DEG.Rdata')
D_edge<-DEG_edgeR[which(DEG_edgeR$change!='NOT'),]
D_limma<-DEG_limmavoom[which(DEG_limmavoom$change!='NOT'),]
D_DES<-DEG_DESeq2[which(DEG_DESeq2$change!='NOT'),]

edgeR<-rownames(D_edge)
dim(D_edge)
limma<-rownames(D_limma)
dim(D_limma)
DESeq2<-rownames(D_DES)
dim(D_DES)

library(VennDiagram)
venn.diagram(
  x = list(
    'edgeR(8930)' = edgeR,
    'limma(8857)' = limma,
    'DESeq2(11160)' = DESeq2
  ),
  filename = 'TCGA DEG VN.png',
  col = "black",
  fill = c("dodgerblue", "goldenrod1", "darkorange1"),
  alpha = 0.5,
  cex = 0.8,
```

```
    cat.col = 'black',
    cat.cex = 0.8,
    cat.fontface = "bold",
    margin = 0.05,
    main = "DEG analysis",
    main.cex = 1.2
)


D_DES$gene<-rownames(D_DES)
D_edge$gene<-rownames(D_edge)
D_limma$gene<-rownames(D_limma)
e_D<-merge(x = D_edge,y = D_DES,by = 'gene')
ed_l<-merge(x = e_D,y = D_limma,by = 'gene')
DEGlist<-ed_l[,c(1,15:21)]
fix(DEGlist)
save(DEGlist,file = '5 TCGA DEG_list_7223.Rdata')
write.csv(DEGlist,file = '5 DEG_list_7223.CSV',row.names = FALSE)
```

## 2. Univariate Cox analysis

```
library(ezcox)
meta<-subset(tcga_meta,tcga_meta$OS.time>0)
sample<-meta[,2]
irg_expr<-irg_expr[which(rownames(irg_expr) %in% sample),]
meta<-data.frame(TCGA_clinical[match(rownames(irg_expr),TCGA_clinical$exprID),],irg_expr)

k<-colnames(irg_expr)
res = ezcox(meta,
            covariates = k,
            time = 'OS.time',
            status = 'OS',
            global_method = c("likelihood", "wald", "logrank")
)
unicox<-res
View(unicox)
write.csv(unicox,file = '3 uniCox.csv',row.names = FALSE)
```

## 3. Constructing and validating risk-score system

```
######contructing risk-score system, LASSO model#######

library(glmnet)
library(survival)
load("C:/Fdisk/CA/LGG/2_survival_analysis/TCGA gene_cox fp.Rdata")
unicox<-read.csv('C:/Fdisk/CA/LGG/2_survival_analysis/3 uniCox.csv')
unicox_dif<-unicox[which(unicox$global.pval<0.05),]
uni_gene<-as.character(unicox_dif[,1])
irg_expr<-new_tcga[,c(17:418)]
fix(new_tcga)

x<-as.matrix(irg_expr[,uni_gene])
y<-data.matrix(Surv(new_tcga$OS.time,new_tcga$OS))

fit0 <- glmnet(x, y, family = "cox", alpha = 1,nlambda = 1000)
plot(fit0)
plot(fit0,xvar="lambda", label=TRUE)

cv.fit <- cv.glmnet(x, y,
                    family="cox",
                    maxit = 1000000,
                    alpha=1)
print(cv.fit)
plot(cv.fit)

fit<-glmnet(x, y, alpha = 1, family='cox',lambda=cv.fit$lambda.min)
plot(fit)
coef(fit)

Coefficients <- coef(fit, s = cv.fit$lambda.min)
Active.Index <- which(Coefficients != 0)
Active.Coefficients <- Coefficients[Active.Index]
Active.Index
Active.Coefficients
lasso_gene<-row.names(Coefficients)[Active.Index]
lasso_min<-data.frame(Active.Index,Active.Coefficients,lasso_gene)

save(lasso_min,file = 'lasso_min_gene_coef.Rdata')
save(cv.fit,fit,lasso_gene,file = 'Lasso model_min.Rdata')

## validation of LASSO model ######
lasso.prob <- predict(cv.fit,
```

```
                          newx=x ,
                          s=c(cv.fit$lambda.min,cv.fit$lambda.1se) )
re=cbind(y ,lasso.prob)
head(re)
```

```
#####    timeROC curve    #####

library(survival)
library(survminer)
library(glmnet)
library(timeROC)

new_tcga$fp=as.numeric(lasso.prob[,1])
with(new_tcga,
        ROC <<- timeROC(T=OS.time,
                          delta=OS,
                          marker=fp,
                          cause=1,
                          weighting="marginal",
                          times=c(365,1095,1825),
                          ROC = TRUE,
                          iid = TRUE)
)
plot(ROC,time=1825,col = "blue",add =FALSE)
plot(ROC,time=1095,col = "blue",add =FALSE)
plot(ROC,time=365,col = "blue",add =FALSE)

ROC$AUC
confint(ROC)
{
auc_365 = ROC$AUC[[1]]
auc_1095 = ROC$AUC[[2]]
auc_1825 = ROC$AUC[[3]]
dat<-data.frame(tpr365=ROC$TP[,1],
                fpr365=ROC$FP[,1],
                tpr1095=ROC$TP[,2],
                fpr1095=ROC$FP[,2],
                tpr1825=ROC$TP[,3],
                fpr1825=ROC$FP[,3])
library(ggplot2)
ggplot() +
  geom_line(data = dat,aes(x = fpr365, y = tpr365),color = "blue") +
  geom_line(data = dat,aes(x = fpr1095, y = tpr1095),color = "red")+
  geom_line(data = dat,aes(x = fpr1825, y = tpr1825),color = "orange")+
  geom_line(aes(x=c(0,1),y=c(0,1)),color = "grey")+
  theme_bw()+
  annotate("text",x = .75, y = .25,
           label = paste("AUC of 1 year = ",round(auc_365,2)),color = "blue")+
  annotate("text",x = .75, y = .15,
```

```
                 label = paste("AUC of 3 years = ",round(auc_1095,2)),color = "red")+
    annotate("text",x = .75, y = .05,
                 label = paste("AUC of 5 year = ",round(auc_1825,2)),color = "orange")+
    scale_x_continuous(name    = "fpr")+
    scale_y_continuous(name = "tpr")
}
```

```
#####         risk level and KMplot        ######

cutoff<-median(as.numeric(lasso.prob[,1]))

new_tcga$fp_level<-ifelse(new_tcga$fp >cutoff,'high','low')
table(new_tcga$fp_level)

fit.surv <-Surv(time = new_tcga$OS.time,event = new_tcga$OS)
km<-survfit(fit.surv~1,data = new_tcga)
km_2<- survfit(fit.surv~fp_level,data=new_tcga)

dat.survdiff=survdiff(Surv(OS.time,OS)~fp_level,data=new_tcga)
p.val = 1 - pchisq(dat.survdiff$chisq, length(dat.survdiff$n) - 1)

ggsurvplot(km_2,
           legend = "top",
           legend.title = 'Risk_score',
           legend.labs = c("High", "Low"),
           linetype = "strata",
           xlab="Days",
           ylab="OS",
           pval=TRUE,
           #conf.int = T,
           risk.table = T,
           risk.table.col= 'strata',
           #palette=c('E7B800','#2E9FDF','#00AFBB')
)
```

####### Risk score analysis, survival analysis and prognostic performance of a riskscore model based on differential expression genes #######

```
library(cowplot)
library(pheatmap)
fp_dat=data.frame(s=1:length(lasso.prob[,1]),v=as.numeric(sort(lasso.prob[,1])))
fp_dat$riskgroup= ifelse(fp_dat$v>cutoff,'high','low')

sur_dat=data.frame(s=1:length(lasso.prob[,1]),
                   t=new_tcga[names(sort(lasso.prob[,1])),'OS.time'] ,
                   e=new_tcga[names(sort(lasso.prob[,1] )),'OS']  )

sur_dat$e=ifelse(sur_dat$e==0,'alive','death')
sur_dat$e=factor(sur_dat$e,levels = c("death","alive"))

exp_dat=new_tcga[names(sort(lasso.prob[,1])),lasso_gene]
{
#### distribution of risk score ####
plot.point=ggplot(fp_dat,aes(x=s,y=v))+
  geom_point(aes(color=riskgroup))+
  #scale_colour_manual(values = c("red","green"))+
  theme_bw()+labs(x="Patient ID(increasing risk score)",y="Risk score")+
  geom_hline(yintercept=cutoff,     #median(fp_dat$v),
             colour="black", linetype="dotted",size=0.8)+
  geom_vline(xintercept=sum(fp_dat$riskgroup=="low"),colour="black",
linetype="dotted",size=0.8)
print(plot.point)

##### distribution of survival time #####
plot.sur<-ggplot(sur_dat,aes(x=s,y=t))+
  geom_point(aes(col=e))+
  theme_bw()+
  #scale_colour_manual(values = c("red","green"))+
  labs(x="Patient ID(increasing risk score)",y="Survival time(year)")+
  geom_vline(xintercept=sum(fp_dat$riskgroup=="low"),colour="black",
linetype="dotted",size=0.8)
print(plot.sur)

##### heatmap of genes expression levels ####
library(pheatmap)
mycolors <- colorRampPalette(c("blue", "white", "red"), bias = 1.2)(100)
tmp=t(scale(exp_dat))
#tmp<-dat_test[,lasso_gene]
#tmp<-t(scale(tmp))
```

```
tmp[tmp > 1] = 1
tmp[tmp < -1] = -1
plot.h=pheatmap(tmp,
                show_rownames = T,
                show_colnames = F,
                legend = F,
                col= mycolors,
                cluster_cols = F
                #cluster_rows = F
)


library(cowplot)
plot_grid(plot.point, plot.sur, plot.h$gtable,
          labels = c("A", "B","C"),
          align = 'v',ncol = 1)
}
```

```
#####    multiple COX regression #####
# univariate Cox analysis
library(ezcox)
new_dat_cox<-new_tcga
new_dat_cox$gender<-as.character(new_dat_cox$gender)    #1 femeal,2 male
new_dat_cox$grade<-ifelse(new_dat_cox$grade == 'G2',2,
                          ifelse(new_dat_cox$grade=='G3',3,'NA'))
new_dat_cox$grade<-as.numeric(new_dat_cox$grade)
new_dat_cox$grade<-as.character(new_dat_cox$grade)

new_dat_cox$codel<-as.factor(new_dat_cox$codel)
new_dat_cox$IDHmut<-as.factor(new_dat_cox$IDHmut)
new_dat_cox$MGMT_methy<-as.factor(new_dat_cox$MGMT_methy)

new_dat_cox$age_level<-ifelse(new_dat_cox$age >40,'high','low')
new_dat_cox$KPS_level<-ifelse(new_dat_cox$KPS >80,'high','low')

new_dat_cox$age_level2<-ifelse(new_dat_cox$age_level=='high','1','0')
new_dat_cox$fp_level2<-ifelse(new_dat_cox$fp_level=='high','1','0')

k<-
c('gender','age','age_level2','grade','KPS_level','KPS','IDHmut','codel','MGMT_methy','fp','fp_le
vel2')
res = ezcox(new_dat_cox,
            covariates = k,
            time = 'OS.time',
            status = 'OS',
            global_method = c("likelihood", "wald", "logrank")
)
write.csv(res,'TCGA 5 clinical_uniCOX.csv',row.names = FALSE)

# multiple Cox regession analysis
multi<-c('age_level2','grade','IDHmut','codel','MGMT_methy','fp_level2')
formula_multicox<-as.formula(paste0('Surv(OS.time,OS)~',
                          paste(multi,sep = '',collapse = '+')))
multi_cox<-coxph(formula_multicox,data = new_dat_cox)
summary(multi_cox)
```

## 4. Development and evaluation of the Nomogram

```
library(rms)

dd<-datadist(new_dat_cox)
options(datadist="dd")
nom_cox<-ph(Surv(OS.time,OS)~age_level2+grade+IDHmut+MGMT_methy+fp_level2,data
= new_dat_cox, x = T,y = T,surv = T)
{
  survival <- Survival(nom_cox)
  survival1 <- function(x)survival(365,x)
  survival3 <- function(x)survival(1095,x)
  survival5 <- function(x)survival(1825,x)
  nom <- nomogram(nom_cox, lp = F ,
                  fun = list(survival1,survival3,survival5) ,
                  fun.at = c(0.1,seq(0.1,0.9,by = 0.2), 0.9),
                  funlabel = c("1-year survival",'3-year survival','5-year survival'))
  plot(nom)
}

# 1-year calibration curve
coxm_1 <- cph(Surv(OS.time,OS)~age_level2+grade+IDHmut+MGMT_methy+fp_level2,
              data=new_dat_cox,
              surv=T,x=T,y=T,
              time.inc = 365)

cal_1<-calibrate(coxm_1,
                 u=365,
                 cmethod='KM',
                 m=80,
                 B=1000)
par(mar=c(7,4,4,3),cex=1.0)
plot(cal_1,lwd=2,lty=1,
     errbar.col=c(rgb(0,118,192,maxColorValue = 255)),
     xlab='Nomogram-Predicted Probability of 1-year OS',
     ylab='Actual 1-year OS(proportion)',
     col=c(rgb(192,98,83,maxColorValue = 255)),
     xlim = c(0,1),ylim = c(0,1))

# 3-year calibration curve
coxm_3 <- cph(Surv(OS.time,OS)~age_level2+grade+IDHmut+MGMT_methy+fp_level2,
              data=new_dat_cox,
              surv=T,x=T,y=T,
              time.inc = 365*3)
```

```
cal_3<-calibrate(coxm_3, u=365*3, cmethod='KM',m=100,B=1000)
plot(cal_3,lwd=2,lty=1,
        errbar.col=c(rgb(0,118,192,maxColorValue = 255)),
        xlab='Nomogram-Predicted Probability of 3-year OS',
        ylab='Actual 3-year OS(proportion)',
        col=c(rgb(192,98,83,maxColorValue = 255)),
        xlim = c(0,1),ylim = c(0,1))

# 5-year calibration curve
coxm_5 <- cph(Surv(OS.time,OS)~age_level+grade+IDHmut+MGMT_methy+fp_level,
                    data=new_dat_cox,
                    surv=T,x=T,y=T,
                    time.inc = 365*5)
cal_5<-calibrate(coxm_5, u=365*5, cmethod='KM',m=100,B=1000)
plot(cal_5,lwd=2,lty=1,
        errbar.col=c(rgb(0,118,192,maxColorValue = 255)),
        xlab='Nomogram-Predicted Probability of 5-year OS',
        ylab='Actual 5-year OS(proportion)',
        col=c(rgb(192,98,83,maxColorValue = 255)),
        xlim = c(0,1),ylim = c(0,1))

# C-index
f <-
coxph(Surv(OS.time,OS)~age_level2+grade+IDHmut+MGMT_methy+fp_level2,data=new_d
at_cox)
sum.surv<-summary(f)
c_index<-sum.surv$concordance
c_index

# timeROC for nomogram model
pred_nom<-predict(f,newdata=new_dat_cox)
new_dat_cox$pred_nom<-as.numeric(pred_nom)
with(new_dat_cox,
        ROC <<- timeROC(T=OS.time,
                              delta=OS,
                              marker=pred_nom,
                              cause=1,
                              weighting="marginal",
                              times=c(365,1095,1825),
                              ROC = TRUE,
                              iid = TRUE)
)
ROC$AUC
confint(ROC)
```

```
{
  auc_365 = ROC$AUC[[1]]
  auc_1095 = ROC$AUC[[2]]
  auc_1825 = ROC$AUC[[3]]
  dat<-data.frame(tpr365=ROC$TP[,1],
                  fpr365=ROC$FP[,1],
                  tpr1095=ROC$TP[,2],
                  fpr1095=ROC$FP[,2],
                  tpr1825=ROC$TP[,3],
                  fpr1825=ROC$FP[,3])
  library(ggplot2)
  ggplot() +
    geom_line(data = dat,aes(x = fpr365, y = tpr365),color = "blue") +
    geom_line(data = dat,aes(x = fpr1095, y = tpr1095),color = "red")+
    geom_line(data = dat,aes(x = fpr1825, y = tpr1825),color = "orange")+
    geom_line(aes(x=c(0,1),y=c(0,1)),color = "grey")+
    theme_bw()+
    annotate("text",x = .75, y = .25,
             label = paste("AUC of 1 year = ",round(auc_365,2)),color = "blue")+
    annotate("text",x = .75, y = .15,
             label = paste("AUC of 3 years = ",round(auc_1095,2)),color = "red")+
    annotate("text",x = .75, y = .05,
             label = paste("AUC of 5 year = ",round(auc_1825,2)),color = "orange")+
    scale_x_continuous(name  = "fpr")+
    scale_y_continuous(name = "tpr")
}


new_tcga_nom<-new_dat_cox
save(new_tcga_nom,file = 'TCGA dataform_age 40.Rdata')
```

## 5. Stratified Analysis

```
# expression levels of identified genes
library(ggpubr)
View(new_tcga_nom)
dat<-new_tcga_nom

tmp1<-subset(dat,dat$histological_type=='Astrocytoma')
tmp1<-data.frame(tmp1$histological_type,tmp1$fp)
colnames(tmp1)<-c('histological_type','risk_score')

tmp2<-subset(dat,dat$histological_type=='Oligoastrocytoma')
tmp2<-data.frame(tmp2$histological_type,tmp2$fp)
colnames(tmp2)<-c('histological_type','risk_score')

tmp3<-subset(dat,dat$histological_type=='Oligodendroglioma')
tmp3<-data.frame(tmp3$histological_type,tmp3$fp)
colnames(tmp3)<-c('histological_type','risk_score')

dat_t<-rbind(tmp2,tmp3)

p1 = ggboxplot(dat, x = "age_level2", y = "fp",
                         color = "age_level2", palette = "jco",
                         add = "jitter")+ stat_compare_means()

p1


# KM plot
library(survival)
library("survminer")

dat<-new_tcga_nom
meta<-dat[which(dat$IDHmut=='0'),]
meta<-dat[which(dat$codel=='1'),]

fit.surv <-Surv(time = meta$OS.time,event = meta$OS)
km<-survfit(fit.surv~1,data = meta)
km_2<- survfit(fit.surv~fp_level,data=meta)
ggsurvplot(km_2,
           legend = "top",
           legend.title = '1p19q_codel',
           legend.labs = c("High", "Low"),
           # risk.table = TRUE,
```

```
  linetype = "strata",
  xlab="Days",
  ylab="OS",
  #palette = c("#E7B800", "#2E9FDF"),
  pval=TRUE
)
```
